# Impact of a poultry education program on elementary students’ knowledge and interest

**DOI:** 10.1016/j.psj.2025.106077

**Published:** 2025-11-07

**Authors:** Dea M Bogdanic, Sara Cloft, Elizabeth L. Karcher

**Affiliations:** Department of Animal Sciences, Purdue University, West Lafayette, IN, 47907, USA

**Keywords:** Agricultural literacy, Elementary student, Poultry science education

## Abstract

The Poultry and Animal Virus Education (PAVE) program was designed to increase agricultural literacy, content knowledge, and science interest among elementary students through a combination of online modules and a hands-on classroom project. A total of 111 students completed baseline surveys (T1), with 78 completing post-module assessments (T2) and 49 completing post-project assessments (T3). Agricultural literacy, module-specific knowledge items, and individual and situational interest were measured using validated Likert-scale instruments. Content knowledge scores remained stable (T1: 6.64 ± 0.21; T2: 6.67 ± 0.49). Individual interest remained neutral between T1 and T3, whereas situational interest increased modestly and remained stable. Students who self-reported low agricultural knowledge scored significantly lower on content knowledge measures (*p* < 0.05). These findings suggest that online interventions may support content understanding but have limited impact on shifting individual interest, highlighting the need to integrate reflection, relevance, and teacher facilitation to sustain engagement.

## Introduction

Agricultural literacy refers to one’s understanding of the food system along with its historical, economic, social, and environmental significance (National Research Council, 1998). As agricultural systems evolve and adapt with new sciences, technologies, and a growing push for sustainability, agricultural literacy is more important now than ever (Cosby et al., 2022). Despite efforts towards educational reform and outreach, research continues to report low levels of agricultural literacy in the United States (Hancock et al., 2024). Misconceptions about agriculture continue to persist, particularly among urban populations, which leads to a lack of understanding that affects consumer behavior, policies, and environmental decision-making (Cosby et al., 2022; Judd-Murray et al., 2024). These misconceptions can also shape consumer perceptions of poultry production, leading to confusion about food safety, animal welfare, and production transparency, which can influence purchasing decisions and public support for agricultural practices (Cosby et al., 2022; Erickson et al., 2019). This is particularly relevant for the poultry industry, where awareness of production practices, biosecurity, and career pathways are minimal among elementary and secondary students (Erickson et al., 2019). Increasing agricultural literacy plays an important role in addressing workforce shortages in agriculture-related fields while also promoting sustainable food systems (Judd-Murray et al., 2024). In youth animal-science contexts specifically, integrated poultry-science curricula have demonstrated measurable gains in agricultural literacy and short-term engagement (Erickson et al., 2019; Marks and Karcher, 2021; Marks et al., 2021).

Perceptions about agriculture often form at a young age, and when students are not taught about food and farming systems, gaps in understanding can persist into adulthood (Cosby et al., 2022). Introducing agricultural concepts to younger students can lead to long-term knowledge gains and stronger connections to food systems and environmental issues (Vallera and Bodzin, 2020; Longhurst and Murray, 2021). Pense et al. (2005) reported that, following the Agriculture in the Classroom intervention, elementary students exposed to agricultural content scored significantly higher on posttest agricultural literacy assessments than their peers in the control group. Similarly, Longhurst and Murray (2021) found that structured, age-appropriate lessons significantly improved agricultural literacy levels in younger students, suggesting that elementary-aged children are highly responsive to these interventions. Elementary-focused poultry programs similarly report sizable immediate gains when lessons are age-appropriate and contextualized (Marks and Karcher, 2021; Marks et al., 2021).

Even with these benefits, agricultural education remains underrepresented at the elementary level due to several challenges. Limited instructional time, pressures over standardized tests, and a general lack of teacher familiarity with agricultural content makes it difficult to incorporate agriculture into early educational settings (Vallera and Bodzin, 2020). In their systematic review, Cosby et al. (2022) similarly highlights that integrating programs with existing classroom structures, conducting long-term impact evaluations, and dealing with lack of resources from schools were among the biggest issues. Recent studies working with an elementary turkey-science curriculum further shows that teacher self-efficacy and implementation constraints significantly shaped both student situational interest and the feasibility of program delivery within classroom settings (Simmermeyer et al., 2022, 2023). These findings point to a clear need for agricultural literacy programs that are accessible, age-appropriate, and designed to fit within the constraints of classrooms. Unlike previous poultry-science curricula that relied on synchronous, teacher-delivered lessons, the PAVE program employed an asynchonous online delivery model that allowed students to progress at their own pace using interactive digital modules paired with a physical notebook and a class project. This hybrid format provided greater implementation flexibility for teachers.

To address these needs, the Poultry and Animal Virus Education (PAVE) program was developed for 4th and 5th grade classrooms. Building on prior poultry-education programs that improved literacy but showed mixed effects on sustained interest (Erickson et al., 2019; Marks et al., 2021), the present study examines whether PAVE influences elementary students’ agricultural literacy, poultry knowledge, and interest. The research questions were:

1) Does participation in the PAVE program affect student agricultural literacy and poultry content knowledge?
2) Does participation in the PAVE program impact student situational and individual interest in animal health and poultry science?

## Methods

### Program development

The elementary Poultry and Animal Virus Education (PAVE) program was created to align with Indiana and Michigan Academic Standards and the National Agriculture Literacy Outcomes (Table 1). The program also intentionally integrated STEM concepts throughout. As part of the program’s development, an advisory committee consisting of two poultry industry representatives and two 4th/5th grade teachers reviewed the curriculum. Suggestions and edits were implemented into the final program. The PAVE program consisted of five online modules, an interactive notebook, two simulation games, and a class project. We asked teachers to implement the program across six consecutive school days, with the expectation that each module would take approximately 30 minutes to complete. The modules were created to be asynchronous, and students completed each independently at their own pace during designated class time under teacher supervision. Thus, the modules were primarily student-directed within a teacher-facilitated environment. On day six, teachers led the class through a class project.

**Table 1:** PAVE program learning outcomes.

Module Title	Learning Outcomes
1: Introduction to Animal Health	Define animal health as it relates to both livestock animals and any animal in your life Identify sick versus healthy animals from visual resources Define One Health and discuss the connection between the health of people, animals, and the environment
2: Production- Farm to Fork	Identify biosecurity issues and define why they are important Relate common health issues and diseases with biosecurity protocols Develop proper practices to keep humans and animals safe
3: Introduction to Animal Immunity	Explain the animal’s defense system and how they fight disease Identify various pathogens and describe how the body protects itself from viruses, bacteria, and fungi
4: Introduction to Vaccinations	Describe the purpose and use of a vaccine Discuss technology used to vaccinate poultry flocks Recognize reasons that vaccines may fail
5: Introduction to the Poultry Industry	Discover different opportunities in animal science and the animal health industry and why they are important Discuss the importance of the poultry industry to Midwest economy (versus United States) Identify how students can become involved with the industry

*Note.* Each online module in the PAVE program was based on learning objectives that were based on the Indiana Academic Standards, Michigan Academic Standards, and National Agricultural Literacy Outcomes for 4th and 5th grade students.

### Context and participants

Elementary school teachers from Michigan and Indiana were recruited to participate in the PAVE program. All participating schools were public, representing both town and rural settings with class sizes ranging from approximately 15 to 21 students. Multiple recruitment methods were utilized and included direct emails to school principals, and the use of listservs. Registration remained open to 4th and 5th grade classrooms on a first-come basis until December 1, 2023. Overall, eight teachers registered for the program. Six out of the eight teachers completed the full implementation of both the online modules and the class project, while the other two discontinued prior to the project phase. This represented 191 students across nine classrooms and included 107 students from Indiana and 84 students from Michigan.

Once registered, teachers were mailed materials for the program. This included consent forms, a teacher program guide, interactive student notebooks, and the materials for the class project. Each student used an individual device to access the modules through Brightspace, ensuring full computer availability. Additionally, we met with each teacher individually in January 2024 to review the expectations for the PAVE program, the research objectives, online logistics, and to answer any questions that teacher had about the program. We requested that all teachers complete the program by April 1, 2024.

### Online modules, interactive student notebooks, and class project

**Figure 1:**
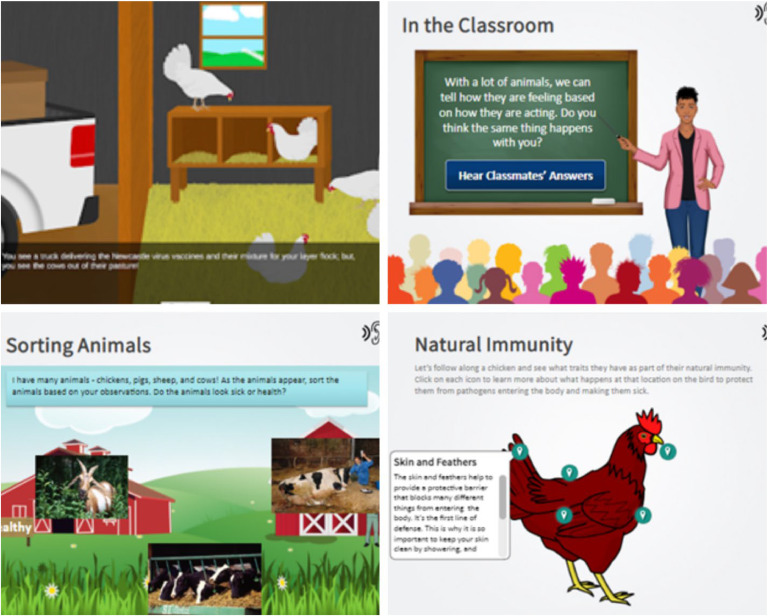
PAVE Program screenshots from online modules.

In order to complete the PAVE program, students received individual login information to access through D2L Brightspace (D2L Corporation, Canada). Modules were created using Story Line 360 software (Articulate, New York, NY). Modules included short videos, animations, and embedded knowledge checks aligned to explicit learning objectives (Fig. 1).

Each student was provided a physical interactive notebook to complete while simultaneously completing the modules. We selected interactive notebooks because they provide students with an opportunity to reflect on their learning and draw connections between the topics and their own experiences (Jaladanki and Bhattacharyya, 2014). The notebooks included a chapter for each module, notes section for the class project, and a final reflection that was completed at the end of the program.

Starting on the sixth day of the PAVE program, students completed a class project. The project included group work and class discussion led by the teacher and focused on biosecurity principles that were presented in the modules. Students used game cards to determine which biosecurity protocols they would use to protect the farm from an invader. Students completed the final reflection question in their interactive notebook immediately following completion of the project.

### Study design

This study was approved by Purdue University’s Institutional Review Board (IRB-2022-1048). A mixed methods approach was used to evaluate the PAVE program’s effectiveness at influencing elementary students’ agricultural literacy, poultry knowledge, and interest. Questionnaires were administered via D2L Brightspace (D2L Corporation, Canada) and assessed student demographics, prior experience with animal agriculture, agricultural literacy, individual interest prior to the program, and situational interest. Prior to the start of the program, teachers provided consent forms and sent consent forms home with students. Both the students and their parents had to consent in order for the student to be included in the data collection portion of the program. One hundred and forty-five students (75.9 %) provided consent and were included in the study. Due to variable responses, only the 5th grade classrooms were included in analysis.

### Instrumentation

Students in the program completed a total of three questionnaires: before beginning module 1 (T1), immediately after completing module 5 (T2) and after the class project (T3). At T1, before beginning the program, students (*n* = 111; 78.55 % response rate) answered seven multiple-choice questions related to demographics and prior agricultural experience. These questions addressed hometown (1 item), experience with different animal species (1 item), participation in agricultural activities (3 items), and knowledge of agriculture and turkey production (2 items). Poultry experience was determined based on students’ responses to items if they had previously owned poultry, participated in poultry-related activities (e.g., 4-H, school projects), or had general exposure to poultry through farm visits or family operations. Students classified their hometown as farm, rural non-farm (<10,000 residents), town (10,000–50,000 residents), suburb (<50,000 residents), or central city (>50,000 residents).

Students completed a baseline elementary agricultural literacy questionnaire (Longhurst Murray Agricultural Literacy Instrument) aligned to the National Agricultural Literacy Outcomes at T1 (22 questions) (Longhurst et al., 2020). Items covered food systems, natural resources, and basic production concepts appropriate for the age group. Response formats included multiple choice and multi-select. Module-specific content knowledge items were researcher-developed to align with PAVE learning objectives and reviewed by two teachers and two poultry-industry representatives to establish content validity (α = 0.83).

Individual interest was assessed with the Individual Interest Questionnaire (IIQ) (α = 0.86-0.90) (Linnenbrink-Garcia et al., 2010) to determine student interest in the poultry industry at T1 and T3 (*n* = 49). Students were provided with five statements relating to their feelings and attitudes towards the poultry industry: the poultry industry is useful for me to know about; the poultry industry helps me in my daily life outside of school; I enjoy learning about the poultry industry; I like the poultry industry; and the poultry industry is exciting to me. Students ranked each statement by selecting one of five emoji faces that corresponded to how they felt about the statement. The individual interest score was then determined by averaging the response of all five questions.

Student situational interest was measured at T2 (*n* = 78; 70.3 %) and T3 (*n* = 49; 44.1 %) using the Situational Interest Scale (SIS; Sun et al., 2008). The SIS consists of 15 Likert-type questions across five subscales: attention demand, challenge, novelty, exploration intention, and instant enjoyment. The Cronbach’s alpha coefficient for intrinsic motivation, identified regulation, external regulation, and amotivation subscales of the SIS were ≥ 0.4 for all subscales.

### Statistical analyses

Statistical analyses were conducted in JMP Pro v16 software. Comparison of scores between timepoints was conducted through z-tests. Comparisons between demographic groups, were conducted through a one-way analysis of variance using only the T1 timepoint responses where demographic questions were asked.

## Results and discussion

### Participant overview

At the start of the program, a total of 111 fifth-grade students across four schools completed the initial PAVE program survey (T1). Participation remained relatively strong at the second timepoint (T2), with 78 responses collected following the completion of the online module (70.3 % response rate). At T3, which was given after the classroom project, 49 students responded (44.1 % response rate). Participation varied by school, with students at School 1 not continuing to respond beyond T1 and other schools showing varying response rates across timepoints. This kind of attrition is consistent with what has been observed in other school-based prevention studies, where factors like student mobility, competing school priorities, and teacher pressure to meet core academic standards can make it difficult to keep the same students engaged (Henneberger et al., 2023). Henneberger et al. also noted that attrition rates in elementary school studies can reach as high as 54 %, particularly in urban schools.

Demographic data from Table 2 indicated that most students came from town or rural areas (82.8 %), with relatively few from farms (7.2 %) or urban settings (5.4 %). Over half (53.5 %) reported no prior poultry exposure, while only a small proportion participated in 4-H (6.3 %). This data suggests that the majority of participants had limited direct agricultural experience, highlighting the relevance of programs like PAVE for introducing agricultural concepts in school-based contexts.

**Table 2:** Participant demographics at baseline (T1).

Demographic Variable	Category	Percentage
Residence Type	Town	43.2 %
	Rural	39.6 %
	Farm	7.2 %
	Suburb	4.5 %
	Central City	5.4 %
Poultry Experience	None	53.5 %
	General	16.7 %
	Owned	21.9 %
	School	5.3 %
	Unspecified	2.6 %
Poultry Production Knowledge	A Little	62.2 %
	No	23.4 %
	Yes	14.4 %
Agricultural Knowledge	Agree	50.88 %
	Neutral	28.95 %
	Disagree	20.18 %
4-H Participation	No	93.7 %
	Yes	6.3 %

Although attrition was expected in a school-based intervention, the overall participation pattern appeared consistent across classrooms and demographic groups. A total of 111 students completed the baseline survey (T1), 78 completed the post-module survey (T2), and 49 completed the post-project survey (T3). Most attrition occurred at the school level due to scheduling conflicts or incomplete teacher implementation rather than individual withdrawal. While no formal analyses were conducted to test for differences between completers and non-completers, demographic comparisons did not indicate any systematic differences that would suggest bias.

### Module content knowledge

Students were assessed on module-specific content knowledge at T1 and T2 using a ten-item quiz aligned with the online learning materials (Table 3). Scores remained consistent across timepoints, with a mean of 6.64 out of 10 (SE = 0.21) at T1 and 6.67 (SE = 0.49) at T2 (*p* = 0.92). While no significant decline occurred, the lack of improvement suggests limited retention of the content presented in the module. Item-level analyses indicated that some outcomes were more challenging than others. For example, many students struggled with items related to biosecurity concepts, suggesting that specific topics may require reinforcement in future curriculum design.

**Table 3:** Module-specific content knowledge scores by Timepoint*.

Topic	T1 Mean ± SE	T2 Mean ± SE
One Health and Poultry Basics	1.98 ± 0.08	1.81 ± 0.21
Poultry Industry Systems	1.23 ± 0.08	1.25 ± 0.11
Poultry Health and Monitoring	1.10 ± 0.09	1.29 ± 0.17
Biosecurity Practices	0.94 ± 0.08	1.12 ± 0.12
Immunity and Vaccination	1.39 ± 0.09	1.15 ± 0.15
**Total**	**6.64 ± 0.21**	**6.67 ± 0.49**

⁎ Values represent mean number correct ± SE. Each module had 2 items; maximum score per module = 2. Total maximum score = 10.

This finding contrasts with previous research showing that targeted agricultural learning modules can lead to significant knowledge gains, especially when grounded in experiential learning principles (Arnold et al., 2006; Erickson et al., 2019; Marks et al., 2021). In these studies, content knowledge increased when students were actively engaged in learning activities that allowed them to apply new concepts in meaningful and hands-on ways. In the case of PAVE, students completed each module during a teacher-scheduled class period, working individually at their own pace with teacher supervision. This delivery style may have limited students’ ability to internalize the content. While some teachers may have offered supplemental support, others might have assigned the module as an independent task, leading to inconsistencies in how the module was experienced. The delivery style may have also been influenced by teachers’ own self-efficacy with science instruction. Teachers with lower self-efficacy may feel less confident facilitating unfamiliar content, which can impact how effectively students engage and learn (Vallera and Bodzin, 2016). Although teacher-level predictors were not analyzed, most participating teachers indicated limited prior exposure to poultry/agriculture at baseline, which likely reduced instructional self-efficacy. Consistent poultry-science modules in high school settings have shown moderate-to-large gains on content quizzes (Erickson et al., 2019). In contrast, this elementary-level program showed little improvement. This difference could be explained by the older age of students, the differences in program design, and the greater instructional support provided in the high school setting. Elementary students likely need more support and hands-on reinforcement to retain the material.

Performance varied based on module content. At T1, the number of items answered correctly were highest for Module 1 (1.98 ± 0.08) and lowest for Module 4 (0.94 ± 0.08). At T2, Module 1 remained among the highest (1.81 ± 0.21). This suggests that students found earlier modules easier to master, which may reflect differences in topic difficulty, alignment with prior knowledge, or how the modules were presented. Across schools, there were no significant differences; however, self-reported agricultural knowledge beliefs were associated with quiz scores. Students who disagreed that they had agricultural knowledge scored significantly lower (mean = 5.61 out of 10) than those who agreed or were neutral (mean = 6.88; *p* < 0.05). These findings suggest that while the content was accessible to some students, the format or structure may not have been equally effective for all learners. Implementation differences across schools may have further impacted student’s outcomes.

### Agricultural literacy scores

Agricultural literacy is defined as the knowledge, skills, and attitudes needed to make informed decisions about agriculture’s role in society (Frick et al., 1991; Vallera and Bodzin, 2016). These items were assessed in this study to evaluate students’ understanding of agriculture across key content areas including food production, environmental topics, and agriculture’s impact on daily life. When asked about their perceived baseline agricultural knowledge, 50.88 % of students agreed they had at least some knowledge, 28.95 % were neutral, and 20.18 % disagreed (Table 2). At T1, of all demographic variables tested, students’ agricultural knowledge belief emerged as the only significant predictor of learning outcomes in both agricultural literacy (16.18 ± 0.44; *p* = 0.0339) and module content knowledge scores (6.88 ± 0.30; *p* = 0.0389). Students who agreed they had some agricultural knowledge achieved higher mean scores than those who were neutral (6.97 ± 0.39) or disagreed (5.61 ± 0.39). Neither poultry experience, 4-H participation, nor production knowledge showed significant associations with student learning outcomes which aligns with prior work suggesting that students’ self-perceived competence can influence academic outcomes more strongly than background experience alone (Bandura, 1997; McKim and Velez, 2016). Students who believed they had agricultural knowledge may have entered the program with greater confidence, which has been linked to increased engagement and persistence in learning tasks. Additionally, this supports existing agricultural literacy models that emphasize the importance of attitudes and beliefs alongside content knowledge and skills (Vallera and Bodzin, 2016).

While the PAVE modules were designed to improve content understanding, this was not observed in our study. Several factors may have contributed to this trend. Notably, the PAVE modules were completed during teacher-scheduled class periods and the time lapses between module completion and the post-survey varied across the classrooms, ranging from approximately four to ten days, with scheduling differences due to snow days, testing, or computer-lab access. Structured agricultural interventions typically result in measurable literacy gains (Pense et al., 2005; Longhurst and Murray, 2021). The decline that was observed in this study may reflect issues related to the implementation, timing, or the challenges teachers faced incorporating the module alongside existing curricular demands, especially if they were unfamiliar with the agricultural content. As noted by Simmermeyer et al. (2023), limited teacher familiarity with agricultural terminology can make it more difficult for students to connect new concepts early in a program, which may temporarily influence how confident they feel about their understanding. Alternatively, agricultural literacy assessments often rely on factual recall, which requires reinforcement of the material. Due to this program spanning only for six days, students may not have had sufficient opportunities to revisit key concepts. This interpretation aligns with findings that, in comparable elementary agriculture contexts, teachers reported low self-efficacy in poultry content knowledge but higher engagement self-efficacy (Simmermeyer et al., 2022, 2023). Another important consideration is that agricultural literacy goes beyond recalling facts. According to Vallera and Bodzin (2016) agricultural literacy encompasses knowledge, skills, attitudes, and beliefs, reflecting not only what students know but also how they apply that knowledge and the values they hold about agriculture. Future studies may benefit from having more open-ended assessments to more accurately capture the full experience of student learning.

### Interest (individual and situational)

Individual interest refers to a student’s long-term engagement with a subject or topic, coupled with consistent curiosity, value, and personal connection (Hidi and Renninger, 2006). Unlike short-term curiosity, individual interest develops gradually through repeated exposure, perceptions of relevance to the learner, and opportunities for reflection. In science education, strong individual interest is linked to improved academic motivation, persistence in STEM, and deeper understanding of the concepts (Hidi and Renninger, 2006; Palmer, 2009).

Individual interest was measured at T1 and T3 only. Mean scores remained neutral, with 2.93 ± 1.1 at T1 and 2.78 ± 1.2 at T3 (*p* = 0.3449). The limited number of responses at T3 (*n* = 49) limits the strength of conclusions that can be drawn. Given that individual interest typically develops gradually through repeated, long-term exposure (Hidi and Renninger, 2006), it may not be reasonable to expect measurable changes within the short duration of the PAVE program. These results suggest that the PAVE program did not significantly impact students’ long-term personal interest in poultry despite exposure to the module.

Several factors may explain these findings. First, the program was administered outside the structure of a formal science curriculum over a period of six days. Without reinforcement, students may have struggled to find the content as personally meaningful. This aligns with Hidi and Renninger’s (2006) findings that situational exposure must be supported through sustained exposure and reflection in order to develop into individual interest. Due to the neutral individual interest, the disconnect may have influenced how students responded. Students may not have recognized the relevance of the questions. Students are less likely to report strong interest when an intervention feels disconnected from their personal goals (Palmer, 2009; Henneberger et al., 2023).

Finally, it is possible that the module did not meet student expectations. Students who already had experience with poultry may have found the online module less engaging or authentic, as the content might have felt repetitive or too basic compared to their prior knowledge (Cloft et al., 2025). This disconnect between expectation and experience can limit long-term interest growth, particularly in programs that lack a hands-on component (Hulleman et al., 2010).

No significant demographic variables predicted change in individual interest scores. While the small T3 sample size may have contributed to this finding, it may also suggest that the combination of the online module and the subsequent hands-on class project was not a strong influence of individual-level interest across student subgroups. Similar patterns regarding knowledge gains with limited shifts in enduring interest were observed in a high school poultry program (Erickson et al., 2019) indicating that brief exposure may be insufficient to alter individual interest without reinforcement.

Situational interest is a short-term form of engagement that arises spontaneously in response to specific features of a task, such as novelty, challenge, or relevance (Hidi and Renninger, 2006; Rotgans and Schmidt, 2011). It is often triggered by activities that capture students’ attention and can strengthen learning. However, if the activity is not supported enough or extended, situational interest tends to fade. In science education, situational interest can support deeper curiosity, focus, and willingness to engage with new material, especially when activities are designed to be inquiry-based, interactive, and personally meaningful (Palmer, 2009; Hulleman et al., 2010).

Situational interest was assessed following the module (T2) and again at T3 (Table 4). At T2, students reported moderate levels of attention (2.77 ± 0.09) and novelty (2.83, SE = 0.08), indicating that the module was initially engaging. The perceived challenge was weaker, suggesting that students did not feel particularly tested by the material. By T3, situational interest declined across most subscales which could suggest that students felt less confident in their ability to succeed in similar science-related activities over time. Given that self-efficacy is closely tied to persistence and engagement in STEM fields (Hulleman et al., 2010; Bathgate et al., 2014), this trend may be particularly important when evaluating the long-term impact of these types of interventions.

**Table 4:** Situational Interest Subscales by Timepoint (Likert Scale 1 Strongly Disagree – 5 Strongly Agree; Mean ± SE).

Subscale	T2 *n* = 78	T3 *n* = 49
Instant Enjoyment	2.59 ± 0.08	2.81 ± 0.11
Challenge	2.63 ± 0.09	2.56 ± 0.11
Attention Demand	2.77 ± 0.09	2.87 ± 0.11
Novelty	2.83 ± 0.08	2.89 ± 0.11
Exploration Intention	2.64 ± 0.09	2.73 ± 0.11

The individually completed modules, coupled with minimal teacher facilitation, may have shaped how students perceived the experience, particularly in contrast to the later group-based classroom component. Prior research has shown that situational interest is influenced on structure and instructional support (Palmer, 2009; Rotgans and Schmidt, 2011). Without a curriculum context, students may not have perceived it as relevant, challenging, or connected it to broader scientific contexts. Henneberger et al. (2023) note that short-term interventions often struggle to shift motivation or interest meaningfully.

These findings suggest that while the PAVE modules initially captured students’ attention, it was not sufficient to maintain situational interest or foster long-term engagement. As Renninger (2016) notes, interest is more likely to persist when students see the information as useful, receive social or instructional support, and engage with the material over time.

### Implications

The results of this paper highlight both the strengths and limitations of the Elementary PAVE program. Students demonstrated some improvements in content knowledge, particularly in earlier modules with more foundational material. However, changes in science attitudes, beliefs, and individual interest were limited. Situational interest remained stable between T2 and T3, which may reflect the added benefit of the teacher-led, hands-on classroom activity at T3.

These mixed outcomes may be explained by factors such as how the modules were delivered, teacher facilitation, and the variations of how it was presented across classrooms. Prior research shows that instructional framing, discussion, and integration into existing curriculums are critical for supporting student motivation and engagement (Palmer, 2009; Hulleman et al., 2010; Rotgans and Schmidt, 2011). Without a clear connection to students’ academic goals or real-world applications, the module may not have been perceived as relevant, particularly for those with no prior poultry experience.

While shorter, interventions like the Elementary PAVE program can offer opportunities to introduce students to agricultural concepts, they may fall short in generating meaningful shifts in interest or identity without continued support. In upper elementary settings, students need more teacher facilitation and novel activities (Rotgans and Schmidt, 2011). Novelty can trigger situational interest but usually can fade without a repeated structure (Hidi and Renninger, 2006; Marks and Karcher, 2021; Simmermeyer et al., 2023). Multi-lesson secondary poultry curricula have produced consistent pre-post knowledge gains (Erickson et al., 2019; Marks et al., 2021). Although these programs were designed in ways similar to PAVE, the differences in age group, baseline knowledge, and instructional support may explain why the elementary-level program yielded weaker improvements. Henneberger et al. (2023) emphasizes that motivation is shaped by repeating, reinforcing experiences. Future iterations may benefit from embedding the modules more deeply, incorporating teacher-guided discussions, and including more hands-on components to help spark and sustain interest. Incorporating design elements used in successful elementary poultry programs like interactive notebooks, team projects, and teacher facilitation, may strengthen both literacy and engagement (Marks and Karcher, 2021; Marks et al., 2021) alongside targeted professional development to bolster teacher self-efficacy (Simmermeyer et al., 2022, 2023).

These findings suggest that brief interventions can support skill confidence and introduce new science content, emphasizing relevance, reflection, and connecting to students’ lived experiences may be key to fostering deeper engagement and sustained interest. For educators, the PAVE model demonstrates how asynchronous digital content paired with reflective notebooks can offer a feasible model for integrating agricultural concepts into elementary classrooms. This structure could be adapted for additional topics, providing a framework for teachers to introduce agriculture in a way that complements learning objectives with classroom schedules.

## Conclusion

The elementary PAVE program demonstrates the potential challenges of introducing agricultural literacy at the upper-elementary level. Students showed some improvement in content knowledge, especially in the foundational modules, but broader shifts in science attitudes and personal interest were limited. Self-perceptions of agricultural knowledge emerged as a consistent predictor of outcomes. These findings highlight that while early digital interventions can spark awareness of agriculture, sustained impact requires developmentally appropriate design, strong teacher facilitation, and reinforcement over time.

## CRediT authorship contribution statement

**Dea M Bogdanic:** Writing – review & editing, Writing – original draft. **Sara Cloft:** Writing – review & editing, Formal analysis. **Elizabeth L. Karcher:** Project administration, Methodology, Investigation, Funding acquisition, Data curation, Conceptualization.

## Disclosures

All authors on the manuscript “Impact of a Poultry Education Program on Elementary Students’ Knowledge and Interest” (Dea Bogdanic, Sara Cloft, and Elizabeth Karcher) have no conflicts of interest to declare for this manuscript.
